# Supplementary material for: The genome sequence of the Muslin Footman moth, Nudaria mundana (Linnaeus, 1761)
Source: Wellcome Open Res. 2024 May 21;9:283. [Version 1] doi: 10.12688/wellcomeopenres.22252.1 (PMC11474146; doi:10.12688/wellcomeopenres.22252.1)
Supplement: Peer review history

# Reviewer report: Reviewer response for version 1

**DOI:** 10.21956/wellcomeopenres.24529.r100840

**Published:** 2024-10-14

**Authors:** Ernesto Samacá-Sáenz

**Affiliations:** 1 UNAM, Mexico City, Mexico

In this manuscript, the complete genome of *N. mundana* was sequenced for the first time from a male specimen collected in Wytham Woods, Oxfordshire, as part of the Darwin Tree of Life Project. The genome assembly resulted in a high-quality genome sequence at chromosome level with most of the genome assigned to 31 chromosomal pseudomolecules   The generated sequences and assemblies are currently available at databases.

 The manuscript is well written and the report of the assembled genome is well presented, however its annotation is still missing, and although they claim they will work on it using RNA-Seq data, it is recommended that the authors include this along with the assembly so more information could be used in future research.

 Moreover, the authors mention that they assembled the mitochondrial genome, however there is no any report on its structure or the genes it contains. Also, a reference image of the circular genome including the recovered  protein-coding genes an tRNAS is lacking . It is recommended that the authors include this information, since the software they used generate this as an output.

Are sufficient details of methods and materials provided to allow replication by others?

Partly

Is the rationale for creating the dataset(s) clearly described?

Yes

Are the datasets clearly presented in a useable and accessible format?

Yes

Are the protocols appropriate and is the work technically sound?

Yes

Reviewer Expertise:

Insect genomics, systematics, evolution

I confirm that I have read this submission and believe that I have an appropriate level of expertise to confirm that it is of an acceptable scientific standard, however I have significant reservations, as outlined above.

# Reviewer report: Reviewer response for version 1

**DOI:** 10.21956/wellcomeopenres.24529.r100841

**Published:** 2024-09-26

**Authors:** Christophe Klopp

**Affiliations:** 1 INRAE, Castanet-Tolosan, France

- The article is well written and simple to read.
- It is unclear what a « chromosomally complete genome » is ? The authors should be more factual about their assembly description.
- The genome assembly mat & meth should indicate if the Hi-C data was included the hifiasm assembly. This is good practice and improves haplotype conservation along contigs.
- It is unclear how haplotypic duplications were identified in the contigs before scaffolding. This should be added.

Are sufficient details of methods and materials provided to allow replication by others?

Partly

Is the rationale for creating the dataset(s) clearly described?

Yes

Are the datasets clearly presented in a useable and accessible format?

Yes

Are the protocols appropriate and is the work technically sound?

Partly

Reviewer Expertise:

Genome and transcriptome assembly and annotation.

I confirm that I have read this submission and believe that I have an appropriate level of expertise to confirm that it is of an acceptable scientific standard, however I have significant reservations, as outlined above.

# Reviewer report: Reviewer response for version 1

**DOI:** 10.21956/wellcomeopenres.24529.r97578

**Published:** 2024-09-21

**Authors:** Naveena NL

**Affiliations:** 1 KSNUAHS, Shivamogga, India

In the background section, you may add answers to these questions:-

 What is the economic importance of this insect? This feeds on lichens and may cause ecological imbalance. Further, can we consider this *Nudaria mundana *as a keystone species?

 In the Methods section:- Mention the lat/long in degree, minutes and seconds- the exact location of the specimen collected.

 In specimen collection and identification paragraph, mention as below:-

 Specimen examined: UK: Wytham Woods, Oxfordshire (biological vice-county Berkshire), 51.77N, 1.34E? 2022-07-22, 1♂, Liam Crowley & Finley Hutchinson: Light trap.

 The collected specimen was identified by Finley Hutchinson as Nudaria mundana (specimen ID Ox003044, ToLID ilNudMund1), and preserved on dry ice.--where? at the University of Oxford or Exeter?

 In the background section, you may add answers to these questions:-

 What is the economic importance of this insect? This feeds on lichens and may cause ecological imbalance. Further, can we consider this *Nudaria mundana *as a keystone species?

 In the material section:- Mention the lat/long in degree, minutes and seconds- the exact location of the specimen collected.

 In specimen collection and identification paragraph, mention as below:-

 Specimen examined: UK: Wytham Woods, Oxfordshire (biological vice-county Berkshire), 51.77N, 1.34E? 2022-07-22, 1♂, Liam Crowley & Finley Hutchinson: Light trap.

 The collected specimen was identified by Finley Hutchinson as Nudaria mundana (specimen ID Ox003044, ToLID ilNudMund1), and preserved on dry ice.

Are sufficient details of methods and materials provided to allow replication by others?

Yes

Is the rationale for creating the dataset(s) clearly described?

Yes

Are the datasets clearly presented in a useable and accessible format?

Yes

Are the protocols appropriate and is the work technically sound?

Yes

Reviewer Expertise:

grain entomology, nanotechnology applications in entomology-sensors for detection, pest management

I confirm that I have read this submission and believe that I have an appropriate level of expertise to confirm that it is of an acceptable scientific standard.
